# Posttraumatic mental health establishment of the Tsunami survivors in Thailand

**DOI:** 10.1186/1745-0179-5-11

**Published:** 2009-06-03

**Authors:** Nuntika Thavichachart, Sookjaroen Tangwongchai, Puangsoy Worakul, Buranee Kanchanatawan, Siriluck Suppapitiporn, Atapol Sukoltapirom na Pattalung, Chutima Roomruangwong, Ongart Chareonsook

**Affiliations:** 1 Department of Psychiatry, Faculty of Medicine, Chulalongkorn University, Bangkok, Thailand; 2 Thailand Center of Excellence for Life Sciences, Bangkok, Thailand

## Abstract

The natural disaster known as "the Tsunami" occurred in Andaman coast of Thailand in December 2004, and there had been questions whether it could cause PTSD amongst the population who lives in the affected area and how to avoid PTSD condition to occur.

The purpose of this study is to establish statistical results of psychosocial factors, and their correlation to PTSD and other mental disorders in order to generate the PTSD database. Cross sectional community surveys had been conducted in two phases from the same sampling group, the first phase is concerned with prevalence of PTSD, depression and related factors. Results were collected from 3,133 samples and shows that 33.6% suffered from PTSD, 14.27% with depression and 11.27% suffered from both. The second phase is focused on chronic PTSD and other mental disorders 2,573 samples were collected and only 21.6% were diagnosed with chronic PTSD.

The statistical analysis has identified risks factors that could cause PTSD, and protective actions which could help to prevent PTSD.

## Introduction

At 7.58 am, on December 26, 2004 an earthquake magnitude of 9.0 Righter scales took place toward the North of Sumatra Island, Indonesia. It generated gigantic waves called Tsunami along the coast of Andaman Sea and Indian Ocean and claimed more than 300,000 lives from 11 affected countries including Thailand. The 2004 Tsunami disaster in the 6 provinces of the southern part of Thailand not only claimed thousands of lives and brought extensive destruction to the affected areas, but also left the survivors with mental disorders especially Posttraumatic Stress Disorder (PTSD) and other mental health problems.

PTSD is a psychiatric disorder that results from the experience or witnessing of traumatic or life-threatening events. During the Civil War, a PTSD-like disorder had been referred to as the 'Da Costa's Syndrome' [1]. PTSD has profound psychobiological correlates, which can impair the person's daily life and be life threatening [2,3]. The diagnosis of PTSD describes symptoms that develop in response to exposure to "extreme traumatic stressors involving direct personal experience of an event or witnessing an event" [4]. These events include natural disasters. Symptoms range from re-experiencing the trauma, persistent avoidance of reminders of the event, numbing of responsiveness, and persistent anxiety or hyper-arousal. For a diagnosis of PTSD, these symptoms must be present for more than one month, and must cause clinically significant distress or impairment in functioning [5].

The prevalence of PTSD from disaster are varies ranging from 11% – 31.8%; Kar & Bastia were study prevalence of PTSD in adolescents after a natural disaster found that post-disaster PTSD presentation in adolescents was 26.9% [6]. Fullerton & et al. examined PTSD in exposed disaster workers (the events of Sept. 11, 2001) found 20.4% of subjects[7]. Mills & et al. were explored prevalence of mental disorders and torture among Tibetan refugees and found that the prevalence of PTSD ranged from 11–23% of subjects [8]. Ozen & Ser were determining the frequency of PTSD in a group of search and rescue workers 2 months after the May 2003 Bingol earthquake found that PTSD was diagnosed in 25% of subjects [9]. Guo & et al. were investigated the prevalence of PTSD among professional and non-professional rescue workers involved in the 1999 Chi-Chi Earthquake in Taiwan found that prevalence of PTSD of professional and non-professional rescuers were 19.8% and 31.8% [10].

Not everyone who experiences the Tsunami develops PTSD. The purposes of this study in the first phase were to determine PTSD and other mental disorders among people age over 18 years old in 6 provinces affected by the Tsunami in Thailand by conducting the community survey from directed affected and controlled area in the same province. To determine the risk and resiliency factors for PTSD by comparing a group of volunteer who developed PTSD from Tsunami with a group of individuals exposed to Tsunami who did not develop the disorder.

In the second phase study were to identify subjects diagnosed with chronic PTSD and subjects without chronic PTSD by re-interviewing the same subjects from the first phase with a set of standard structured interviews. We also looked for and interviewed subjects without chronic PTSD, a sib-subjects without chronic PTSD, 18 years or older, and both parents of the first phase subjects diagnosed with PTSD were also included to the study.

## Materials and methods

### Participants

The first phase of the study was collected by cross-sectional community survey data from 3,133 subjects age over 18 years old in direct affected and controlled area of the 6 provinces of Thailand, namely, Ranong, Pang-nga, Phuket, Krabi, Trang, and Satoon.

The second phase study collected 2,573 subjects from the first phase. The subjects were followed and collected the data by well trained interviewers including subject diagnosed with PTSD, subject without PTSD, and the sib of the subject diagnosed with PTSD after being informed and signed the inform consent form including psychiatric treatment follow up.

### Instruments

#### The first phase

- Demographic data & exposure to Tsunami information

- Davidson Trauma Scale, (confirmed by psychiatric interview using CAPSII)

17-item, brief global assessment scale for posttraumatic stress disorder (PTSD).

The DTS showed good internal consistency (Cronbach alpha = .97) and test-retest reliability (r = .88 – .93) [11-13].

- Beck Depression Inventory: BDI

BDI was developed by Beck et al. (1979). BDI is a self-report scale with 21 items that measure the emotional, somatic, cognitive, and motivational symptoms seen in depression. The aim of the scale is to determine the severity of depressive symptoms. Correlation coefficients by split half reliability were 0.74, BDI scores ≥ 20 were reported to discriminate depression that might require treatment with more than 90% accuracy. The score of each item ranges from 0 to 3 [14].

- Symptom Check List 90: SCL 90

SCL 90 is a self-rated scale that consists of eight psychiatric symptom domains.

It consists of 90 items total, nine subscales: somatization, obsessive-compulsive, interpersonal sensitivity, depression, anxiety, hostility, phobic-anxiety, paranoid ideation and psychoticism [15].

- Maudsley Personality Inventory: MPI

MPI is a self-rated scale that consists of 48 items, to indicate scores on the dimensions of extraversion and neuroticism. The correlation between corresponding scales are high, r = .86 for neuroticism and r = .87 for extraversion [16].

- Brief Cope Scale

Brief Cope Scale is a multidimensional coping inventory to assess the different ways in which people respond to stress. Brief Cope Scale consists of 28 items total,14 dimension as follows: Self-distraction, Active coping, Denial, Substance use, Use of emotional support, Use of instrumental support, Behavioral disengagement, Venting, Positive reframing, Planning, Humor, Acceptance, Religion, Self-blame [17,18].

#### The second phase

- Semi-Structured Assessment for Drug Dependence and Alcoholism: SSADDA

SSADDA is a diagnostic instrument developed for studies of the genetics of substance use and associated disorders [19].

- Composite International Diagnostic Interview: CIDI

CIDI is a comprehensive and fully standardized diagnostic interview designed for assessing mental disorders according to the definitions of the Diagnostic Criteria for Research of ICD-10 and DSM-III-R [20,21].

### Procedure

The first phase, Sample frame was multi-stage randomly selected, between February-March, 2005 (within 3 months post-Tsunami). An Informed consent was obtained from all the participants. The samples were interviewed by well trained last-year nurse students, and confirmed by psychiatric interview with CAPS II and also gave the psychiatric treatment by psychiatrists which took approximately 55 minutes to complete the questionnaire.

The second phase, Subjects from the first phase were followed and collected the data by well trained interviewers including subject diagnosed with PTSD, subject without PTSD, and the sib of the subject diagnosed with PTSD after being informed and signed the inform consent form including psychiatric treatment follow up. The second phase was implemented during May-August, 2005 (6 months post-Tsunami).

Descriptive Statistics were used to record the prevalence of PTSD, chronic PTSD & other mental disorders. Chi-square was conducted to determine the relationship between PTSD and other variables. These variables were then included as predictor variables in a Binary logistic regression analysis. All analyses were conducted using SPSS for windows [22].

## Results

### The first phase

The 1,054 (33.60%) was diagnosed as PTSD, 447 (14.27%) was diagnosed as having depression and 353 (11.27%) had both PTSD & depression as a co-morbidity. The prevalence of PTSD, depression and co-morbidity classified by province are show in Table 1.

**Table 1:** The prevalence of PTSD, Depression and Co-Morbidity

Province	PTSD	Depression	Co-Morbidity	Normal
	n (%)	n (%)	n (%)	n (%)
Pang-nga	545 (51.7)	238 (53.2)	194 (55.0)	1,058 (53.3)
Ranong	64 (6.1)	27 (6.0)	23 (6.5)	91 (4.6)
Phuket	71 (6.7)	24 (5.4)	16 (4.5)	161 (8.1)
Krabi	119 (11.3)	42 (9.4)	31 (8.8)	203 (10.2)
Trang	170 (16.1)	72 (16.1)	55 (15.6)	324 (16.3)
Satoon	85 (8.1)	44 (9.8)	34 (9.6)	148 (7.5)

total	1,054 (100)	447 (100)	353 (100)	1,985 (100)

The comparison of characteristic variables between people diagnosed with PTSD and without PTSD which revealed that there was difference between them in their Gender, χ^2 ^(1, *N *= 3,133) = 8.06, *p *< .01, Age, χ^2 ^(3, *N *= 3,133) = 14.53, *p *< .01, Marital Status, χ^2 ^(2, *N *= 3,133) = 6.01, *p *< .05, Educational Status, χ^2 ^(4, *N *= 3,133) = 9.47, *p *< .05, Affected Area, χ^2 ^(1, *N *= 3,133) = 219.11, *p *< .001, Physical Condition, χ^2 ^(1, *N *= 3,133) = 22.79, *p *< .001, Having History of Previous Trauma before the age of twelve, χ^2 ^(1, *N *= 3,133) = 33.24, *p *< .001, Having History of Previous Trauma during 12–18 years, χ^2 ^(1, *N *= 3,133) = 25.71, *p *< .001, Known Tsunami Before, χ^2 ^(1, *N *= 3,133) = 4.83, *p *< .05, Physical Injury, χ^2 ^(1, *N *= 3,133) = 135.25, *p *< .001, Loss of Family Member(s), χ^2 ^(1, *N *= 3,133) = 26.89, *p *< .001, Loss of Property, χ^2 ^(1, *N *= 3,133) = 195.66, *p *< .001, Loss of Career, χ^2 ^(1, *N *= 3,133) = 59.93, *p *< .001 (Table 2)

**Table 2:** The prevalence characteristics of the study sample (n = 3,133)

Characteristics	PTSD (n = 1,054)	Without PTSD (n = 2,079)	statistics	
	n (%)	n (%)	χ^2^	*p*
Gender				
male	414(39.3)	927(44.6)	8.06	.005**
female	640(60.7)	1,152(55.4)		
				
Age				
≤ 28	299(28.4)	543(26.1)	14.53	.002**
29–37	268(25.4)	487(23.4)		
38–48	284(26.9)	522(25.1)		
≥ 49	203(19.3)	527(25.3)		
M = 38.73, SD = 14.14 yrs.				
				
Marital Status				
single	158(15.0)	382(18.4)	6.01	.05*
married	809(76.8)	1,519(73.1)		
divorced/widowed	87(8.2)	178(8.5)		
				
Educational Status				
illiterate	15(1.4)	40(1.9)	9.47	.05*
elementary school	677(64.3)	1,248(60.0)		
primary school	176(16.7)	358(17.2)		
secondary school	151(14.3)	324(15.6)		
academic degree	35(3.3)	109(5.3)		
				
Affected Area				
direct affected area	823(78.1)	1,053(50.6)	219.11	.000**
non direct affected area	231(21.9)	1,026(49.4)		
				
Physical Condition				
illness	308(29.2)	447(21.5)	22.79	.000**
healthy	746(70.8)	1,632(78.5)		
				
History of Previous Trauma, < 12 yrs.				
trauma	482(45.7)	730(35.1)	33.24	.000**
no trauma	572 (54.3)	1,349(64.9)		
				
History of Previous Trauma, 12–18 yrs.				
trauma	419(39.8)	638(30.7)	25.71	.000**
no trauma	635(60.2)	1,441(69.3)		
				
Known Tsunami Before				
known	358(34.0)	626(30.1)	4.83	.028*
unknown	696(66.0)	1,453(69.9)		
				
Physical Injury				
physical injury	327(31.0)	283(13.6)	135.25	.000**
no Physical injury	727(69.0)	1,796(86.4)		
				
Loss of Family Member				
yes	588(55.8)	956(46.0)	26.89	.000**
no	466(44.2)	1,123(54.0)		
				
Loss of Property				
yes	240(22.8)	1,012(48.7)	195.66	.000**
no	814(77.2)	1,067(51.3)		
				
Loss of Career				
yes	493(46.8)	678(32.6)	59.93	.000**
no	561(53.2)	1,401(67.4)		

*p < .05. **p < .01. ***p < .001.

The results from the Beck Depression Inventory-BDI and SCL90 revealed significant differences between those with and without PTSD on the degree of depression (Severe depression > depression > normal), χ^2 ^(2, *N *= 3,133) = 481.12, *p *< .001, & other symptom; Somatization, χ^2^(1, *N *= 3,133) = 229.61, *p *< .001, Obsessive-Compulsive, χ^2 ^(1, *N *= 3,133) = 144.15, *p *< .001, Interpersonal Sensitivity, χ^2 ^(1, *N *= 3,133) = 49.32, *p *< .001, Depression, χ^2 ^(1, *N *= 3,133) = 268.35, *p *< .001, Anxiety, χ^2 ^(1, *N *= 3,133) = 109.54, *p *< .001, Hostility, χ^2 ^(1, *N *= 3,133) = 39.44, *p *< .001, Phobic Anxiety, χ^2 ^(1, *N *= 3,133) = 327.52, *p *< .001, Paranoid Ideation, χ^2 ^(1, *N *= 3,133) = 71.61, *p *< .001, Psychoticism, χ^2 ^(1, *N *= 3,133) = 96.74, *p *< .001 (Table 3)

**Table 3:** The psychological test score by PTSD & Without PTSD (n = 3,133)

Characteristics	PTSD (n = 1,054)	Without PTSD (n = 2,079)	statistics	
	n (%)	n (%)	χ^2^	*p*
Beck Depression Inventory				
Severe Depression	86(8.2)	17(.8)	481.12	.000**
Depression	267(25.3)	77(3.7)		
Usual	701(66.5)	1,985(95.5)		
*M *= 9.32, *SD *= 8.86				
				
Symptom Distress Checklist				
Somatization	208(19.7)	71(3.4)	229.61	.000**
Usual	846(80.3)	2,008(96.6)		
*M *= .53, *SD *= .52				
				
Obsessive-Compulsive	121(11.5)	34(1.6)	144.15	.000**
Usual	933(88.5)	2,045(98.4)		
*M *= .66, *SD *= .61				
				
Interpersonal Sensitivity	51(4.8)	19(0.9)	49.32	.000**
Usual	1,003(95.2)	2,060(99.1)		
*M *= .48, *SD *= .51				
				
Depression	183(17.4)	34(1.6)	268.35	.000**
Usual	871(82.6)	2,045(98.4)		
*M *= .52, *SD *= .54				
				
Anxiety	80(7.6)	16(.8)	109.54	.000**
Usual	974(92.4)	2,063(99.2)		
*M *= .55, *SD *= .57				
				
Hostility	36(3.4)	11(.5)	39.44	.000**
Usual	1,018(96.6)	2,068(99.5)		
*M *= .68, *SD *= .37				
				
Phobic Anxiety	281(26.7)	93(4.5)	327.52	.000**
Usual	773(73.3)	1,986(95.5)		
*M *= .51, *SD *= .57				
				
Paranoid Ideation	60(5.7)	16(.8)	71.61	.000**
Usual	994(94.3)	2,063(99.2)		
*M *= .35, *SD *= .49				
				
Psychoticism	76(7.2)	18(0.9)	96.74	.000**
Usual	978(92.8)	2,061(99.1)		
*M *= .27, *SD *= .38				
				
Brief cope scale				
Active Coping	581(55.12)	768(36.94)	94.31	.000**
Usual	473(44.88)	1,311(63.06)		
*M *= 2.33, *SD *= 1.41				
				
Planning	501(47.53)	572(27.51)	124.48	.000**
Usual	553(52.47)	1,507(72.49)		
*M *= 2.03, *SD *= 1.45				
				
Positive Reframing	522(49.53)	789(37.95)	38.50	.000**
Usual	532(50.47)	1,290(62.05)		
*M *= 2.29, *SD *= 1.47				
				
Acceptance	712(67.55)	1,254(60.32)	38.50	.000**
Usual	342(32.45)	825(39.68)		
*M *= 3.0, *SD *= 1.53				
				
Humor	422(40.04)	691(33.24)	14.12	.000**
Usual	632(59.96)	1,388(66.76)		
*M *= 2.05, *SD *= 1.48				
				
Religion	612(58.06)	979(47.09)	33.70	.000**
Usual	442(41.94)	1,100(52.91)		
*M *= 2.73, *SD *= 1.72				
				
Use of Emotion Support	629(59.68)	828(39.83)	110.78	.000**
Usual	425(40.32)	1,251(60.17)		
*M *= 2.54, *SD *= 1.47				
				
Use of Instrumental Support	477(45.26)	618(29.73)	74.19	.000**
Usual	577(54.74)	1,461(70.27)		
*M *= 2.15, *SD *= 1.40				
				
Self Distraction	631(59.87)	797(38.34)	130.72	.000**
Usual	423(40.13)	1,282(61.66)		
*M *= 2.40, *SD *= 1.54				
				
Denial	257(24.38)	185(8.90)	138.39	.000**
Usual	797(75.62)	1,894(91.10)		
*M *= 1.07, *SD *= 1.28				
				
Venting	482(45.73)	500(24.05)	152.77	.000**
Usual	572(54.27)	1,579(75.95)		
*M *= 2.0, *SD *= 1.36				
				
Substance Use	92(8.73)	105(5.05)	16.06	.000**
Usual	962(91.27)	1,974(94.95)		
*M *= .60, *SD *= 1.12				
				
Behavioral Disengagement	136(12.90)	79(3.79)	90.68	.000**
Usual	918(87.1)	2,000(96.20)		
*M *= .7, *SD *= 1.07				
				
Self Blame	90(8.54)	58(2.79)	51.36	.000**
Usual	964(91.46)	2,021(97.21)		
*M *= .55, *SD *= .96				
				
Maudsley Personality Inventory				
E Scale				
(*M *= 25.72, *SD *= 6.01)				
Introvert	634(60.2)	1,064(51.2)	22.69	.000**
Extrovert	420(39.8)	1,015(48.8)		
				
N Scale (*M *= 20.41, *SD *= 11.51)				
Neurotic	767(72.8)	729(35.1)	398.55	.000**
Stability	287(27.2)	1,350(64.9)		

*p < .05. **p < .01. ***p < .001.

The results from the brief cope scale revealed significant differences using by those with and without PTSD; Active Coping, χ^2 ^(1, *N *= 3,133) = 94.31, *p *< .001, Planning Coping, χ^2 ^(1, *N *= 3,133) = 124.48, *p *< .001, Positive Reframing Coping, χ^2 ^(1, *N *= 3,133) = 38.50, *p *< .001, Acceptance Coping, χ^2^(1, *N *= 3,133) = 38.58, *p *< .001, Humor Coping, χ^2^(1, *N *= 3,133) = 14.12, *p *< .001, Religion Coping, χ^2^(1, *N *= 3,133) = 33.70, *p *< .001, Using Emotion Support Coping, χ^2 ^(1, *N *= 3,133) = 110.78, *p *< .001, Using Instrumental Support Coping, χ^2 ^(1, *N *= 3,133) = 74.19, *p *< .001, Self-Distraction Coping, χ^2 ^(1, *N *= 3,133) = 130.72, *p *< .001, Denial Coping, χ^2 ^(1, *N *= 3,133) = 138.39, *p *< .001, Venting Coping, χ^2 ^(1, *N *= 3,133) = 152.77, *p *< .001, Substance Use Coping, χ^2 ^(1, *N *= 3,133) = 16.06, *p *< .001, Behavioral Disengagement Coping, χ^2^(1, *N *= 3,133) = 90.68, *p *< .001, Self-Blame Coping, χ^2 ^(1, *N *= 3,133) = 51.36, *p *< .001 (Table 3)

The factors suggested by the bivariate analyses that associated with PTSD were entered into a binary logistic regression analysis model. The model identified thirteen independent variables that correctly predicted 76.6% of individuals who developed to PTSD.

Binary Logistic Regression Model for PTSD. The logistic equation is: -3.41 + .95 (Direct Affected Area) + .21(History of Previous Trauma < 12 years) + .50 (Physical Injury) + .12 (Planning Coping) – .13 (Humor Coping) + .93 (Use of Emotion Support Coping) + .94 (Self Distraction Coping) + .19 (Denial Coping) + .10 (Venting Coping) – .08 (Substance Use Coping) + .15 (Behavioral Disengagement Coping) + .07 (Personality: N scale) – .02 (Personality: E scale).

A weighted combination of the thirteen independent variables correctly predicted 76.6% of individuals who developed to PTSD (Table 4).

**Table 4:** Binary Logistic Regression Model of factors associated with PTSD

Characteristics	*B*	*S.E*.	*p*	OR (95%CI)	
Constant	-3.412	.253	-	-	-
Direct affected area	.952	.102	.000	2.59	(2.12–3.16)
Having History of Previous Trauma (< 12 years)	.214	.091	.018	1.24	(1.04–1.48)
Physical injury	.504	.110	.000	1.66	(1.33–2.05)
Planning	.124	.035	.000	1.13	(1.06–1.21)
Humor	-.134	.035	.000	.87	(.82 – .94)
Use of Emotion Support coping	.093	.038	.014	1.09	(1.02–1.18)
Self distraction coping	.094	.037	.011	1.10	(1.02–1.18)
Denial coping	.194	.037	.000	1.21	(1.13–1.31)
Venting coping	.100	.040	.013	1.11	(1.02–1.19)
Substance Use coping	-.083	.040	.039	.92	(.85 – .99)
Behavioral Disengagement coping	.145	.044	.001	1.56	(1.06–1.26)
Personality: N Scale	.068	.004	.000	1.07	(1.06–1.08)
Personality: E Scale	-.021	.008	.007	.98	(.96 – .99)

### The Second phase

Table 5 presents, prevalence of chronic PTSD (from SADDA) & other mental disorders from CIDI, the 556 (21.60%) as chronic PTSD, and 100 (3.9%) having a life time prevalence PTSD, 472 (18.3%) as having Major Depression, 165 (6.4%) as Manic, 214 (8.3%) as Panic, 103 (4.0%) as Simple Phobia, 162 (6.3%) as Agoraphobia, 65 (2.5%) as Generalized Anxiety, 284 (11.0%) as Obsessive-Compulsive, and 26 (1.0%) as Social Phobia.

**Table 5:** Prevalence of chronic PTSD & other mental disorders from CIDI

*Characteristics *(N = 2,573)	Case	Non-case
	
	n (%)	n (%)
Chronic PTSD	556 (21.6)	2,017 (78.4)
Have a life time prevalence PTSD	100 (3.9)	2,473 (96.1)
Patient with mental disorders from CIDI		
1) Mood disorder		
Major depression	472 (18.3)	2,101 (81.7)
Manic	165 (6.4)	2,408 (93.6)
2) Anxiety disorder		
Panic	214 (8.3)	2,359 (91.7)
Simple phobia	103 (4.0)	2,470 (96.0)
Agoraphobia	162 (6.3)	2,411 (93.7)
Generalized anxiety	65 (2.5)	2,508 (97.5)
Obsessive-compulsive	284 (11.0)	2,289 (89.0)
Social phobia	26 (1.0)	2,547 (99.0)
3) Substance use disorder		
Alcohol abuse	515 (20.0)	2,058 (80.0)
Alcohol dependence	139 (5.4)	2,434 (94.6)
Nicotine abuse	363 (14.1)	2,210 (85.9)
Nicotine dependence	44 (1.7)	2,529 (98.3)
Cannabis abuse	44 (1.7)	2,529 (98.3)
Cannabis dependence	16 (.6)	2,557 (99.4)
Amphetamine abuse	40 (1.6)	2,533 (98.4)
Amphetamine dependence	8 (.3)	2,565 (99.7)
Khatom abuse	90 (3.5)	2,483 (96.5)
Khatom dependence	3 (.1)	2,570 (99.9)
Betel abuse	56 (2.2)	2,517 (97.8)
Betel dependence	4 (.2)	2,569 (99.8)

For the substance use disorder: Alcohol Abuse and Dependence were 515 (20%) and 139 (5.4%), Nicotine Abuse and Dependence were 363 (14%) and 44 (1.7%), Cannabis Abuse and Dependence were 44 (1.7%) and 16 (.6%), Amphetamine Abuse and Dependence were 40 (1.6%) and 8 (.3%), Khatom Abuse and Dependence were 90 (3.5%) and 3 (.1%), Betel Abuse and Dependence were 56 (2.2%) and 4 (.2%).

## Discussion

The prevalence of PTSD, Depression, and Co-morbid after the Tsunami were 33.6% 14.3% and 11.3% respectively. PTSD and Depression were the most prevalent disorders after the disaster and showed a decrease over time.

A weighted combination of the thirteen independent variables correctly predicted 76.6% of traumatized individuals who developed PTSD. All predicted variables correlate with psychological factors especially "Direct Affected Area" and "Physical Injury". These variables that explain in part of severity of the traumatic event is considered to be one of the most salient predictors of PTSD [23]. In the part of physical injury variable, Davidson & Smith found that PTSD tended to be caused by physically injured and hospitalized after a traumatic event [24].

The variable "Having History of Previous Trauma before the age of twelve", was significant predictors of PTSD. Having reviewed the effects of childhood trauma as a risk factor for later developing PTSD, Breslau & Chilcoat indicated that who were reported of any previous trauma significantly more likely experienced PTSD than those with no previous exposure to trauma [25].

Regarding, PTSD & Coping, literature suggests that some methods of coping are more effective for some people or some situations, and the way people process and interpret traumatic events and its consequences may play a role in the development or maintenance of PTSD [26,27]. The result from binary logistic analysis of this study shows that some methods of coping, Humor & Substance use, are resilient in PTSD because reciprocal inhibition theory confirms that "two antagonistic responses could not coexist in the same organism" and happiness & sadness could not also coexist in patients at the same time. Some therapists use this theory to develop exposure counter-conditioning technique which is one of the cognitive behavioral therapies. The technique has strong empirical support as the treatments of choice for PTSD [28,29]. Patients consume psychoactive substances to improve their mood or to escape from adverse emotions. The reinforcement received may lead to elevated substance use rates and subsequently the use-related negative consequences that characterize substance abuse. That is a reason many researches find that PTSD symptomatology is significantly associated with alcohol and other substance use. However, there are several negative outcomes that should not be neglected. Many researches found out that the group of PTSD patients with substance use resulted in more other psychiatric problems and total psychiatric symptoms prior to relapse than those without substance use. Thus, this suggests that both PTSD and substance use have a poorer clinical course [30].

PTSD & Personality, predisposing personal characteristics possibility that there may be particular risk factors that make an individual vulnerable towards developing a PTSD. We found that introvert & neurotic personality that associate with PTSD to be in agreement with Aidman & Kollaras-Mitsinikos found that intrusion symptoms were predicted both by Extraversion and Neuroticism but avoidance symptoms was predicted by Neuroticism only [31].

## Conclusion

We finally established a huge clinical data base of mental health problems among Tsunami survivors in Thailand, 2004. We found 1,054 subjects diagnosed with PTSD, 447 with depression and 353 co-morbidity subjects. Among 1,054 subjects diagnosed with PTSD, 556 was diagnosed to chronic PTSD after 6 months. There were psychosocial factors identified as risk and protective factors for PTSD & depression. In comparison between the first phase and the second phase, the present study showed that the prevalence of PTSD was still higher in the affected region 6 months after the Tsunami. Therefore, the researcher team will concentrate on treatment for the patients of chronic PTSD and other mental disorders in the third phase that include analysis blood samples data in the future.

## Competing interests

The authors declare that they have no competing interests.

## Authors' contributions

The work presented here was carried out in collaboration between all authors. NT defined the research theme and participated in the design of the study. ST performed the statistical analysis and interpretation of data. PW carried out the psychological analysis. BK, SS and ASnP participated in the psychiatry and epidemiology study. CR and OC coordinated on the field study and acquisition of data. All authors have contributed to, seen and approved the final manuscript.
